# Supplementary material for: Direct Imaging of Hydrogen‐Driven Dislocation and Strain Field Evolution in a Stainless Steel Grain
Source: Adv Mater. 2025 Sep 9;37(45):e00221. doi: 10.1002/adma.202500221 (PMC12617056; doi:10.1002/adma.202500221)
Supplement: Supplementary file 1 — Supporting Information [file ADMA-37-e00221-s002.pdf]

ADVANCED MATERIALS

Supporting Information

for *Adv. Mater.*, DOI 10.1002/adma.202500221

Direct Imaging of Hydrogen-Driven Dislocation and Strain Field Evolution in a Stainless Steel Grain

David Yang, Mujan Seif, Guanze He, Kay Song, Adrien Morez, Benjamin de Jager, Dmytro Nykypanchuk, Ross J. Harder, Wonsuk Cha, Edmund Tarleton, Ian K. Robinson and Felix Hofmann**

Supporting Information for Direct Imaging of Hydrogen-Driven Dislocation and Strain Field Evolution in a Stainless Steel Grain

David Yang[1,2*], Mujan Seif[1], Guanze He[1†], Kay Song[1§],
Adrien Morez[1], Benjamin de Jager[1], Dmytro Nykypanchuk[3], Ross J. Harder[4],
Wonsuk Cha[4], Edmund Tarleton[1], Ian K. Robinson[2,5], Felix Hofmann[1*]

[1]Department of Engineering Science, University of Oxford, Oxford OX1 3PJ, UK

[2]Condensed Matter Physics and Materials Science Department, Brookhaven National Laboratory, Upton, NY 11973, USA

[3]Center for Functional Nanomaterials, Brookhaven National Laboratory, Upton, NY 11973, USA

[4]Advanced Photon Source, Argonne National Laboratory, Lemont, IL 60439, USA

[5]London Centre for Nanotechnology, University College London, London WC1E 6BT, UK

[†]Present address: Shanghai Nuclear Engineering Research and Design Institute, Shanghai 200233, China.

[§]Present address: School of Aerospace, Mechanical and Mechatronic Engineering, The University of Sydney, Sydney, NSW 2006, Australia.

[*]Corresponding author. Email: david.yang@eng.ox.ac.uk, felix.hofmann@eng.ox.ac.uk

This PDF file includes:

Supporting Text

Figures S1 to S13

Tables S1 and S2

Captions for Videos S1 to S3

Other Supporting Materials for this manuscript:

Videos S1 to S3

Supporting Text

Electrochemical flow cell

Figure S1 shows different views of the electrochemical flow cell. The body of the flow cell was 3D printed using Rigid 10K resin from SGD 3D. Luer locks were attached to the fluid ports and a Thorlabs 1X1 kinematic mount was attached to the opening on the bottom, using epoxy. M3 thread inserts were tapped into the cell body. A piece of chemically resistant double sided tape (from 3M) was attached to the square sample pedestal in the flow cell. A Pt wire was fixed to the top of the tape and threaded through the tape, flow cell and the kinematic mount. The HPT disk was then placed on top of the tape, making contact with the Pt wire, forming the working electrode. The counter electrode was formed using a Pt wire coil threaded through the side of the flow cell, and sealed with epoxy on the outside. The Pt wires were soldered to insulated wires to connect to the SP-300 Biologic potentiostat. A nitrile O-ring (20 mm bore, 23 mm outer diameter) was placed beneath a Kapton thin film which covered the sample. The lid was 3D printed using 3D Systems M2R-TN photopolymer resin (from the Department of Engineering Science at Oxford) and attached using M3 countersunk screws.

BCDI reconstruction amplitude threshold

Having an accurate representation of the 3D grain morphology is crucial for computing the surface strain, which is limited to the size of the voxel. The amplitude threshold was set to 0.20 for the initial reconstruction at –3.4 h. Due to slight fluctuations in the reconstruction convergence, the same amplitude threshold would alter the volume of the reconstructed grain. Assuming the effective grain size for the 111 reflection does not change, the volume of the crystal should be constant. The amplitude threshold was automatically adjusted such that the reconstructions have a similar volume to the initial reconstruction, within a tolerance of 50 voxels. Overall, amplitude thresholds have an average of 0.14 and a standard deviation of 0.034.

Spatial resolution

The 3D spatial resolution was determined to be 12 nm averaged over all reconstructions. This was determined by computing the phase retrieval transfer function (PRTF) [1], which measures the ratio between the reconstructed Fourier magnitude and the actual measured Fourier magnitude across spatial frequencies,

$$\text{PRTF}(\mathbf{Q}) = \frac{|\mathcal{F}[\rho(\mathbf{r})]|}{\sqrt{I^{\text{meas}}(\mathbf{Q})}} \tag{S1}$$

where $\rho(\mathbf{r})$ is the reconstructed complex electron density. The resolution was conservatively determined as the frequency when the PRTF = 0.5 [1] for each reconstruction, though a more liberal estimate is when the PRTF = $1/e$, which would yield an average spatial resolution of 11 nm. An example of using the PRTF to determine the spatial resolution is shown in Figure S5a, with the spatial resolution for each reconstruction shown in Figure S5b.

Orientation matrix calculation

At least two known crystallographic directions must be known to compute the orientation matrix, **UB**. First, we know that $\mathbf{Q}_{hkl}$ points to $[111]$, call this normalised direction $\hat{\mathbf{A}}$ in sample coordinates. Second, the plane that the large dislocation lies on must be $\{111\}$, assuming it is a shear loop expected for FCC metals. Here, we arbitrarily say it is $(\bar{1}11)$. We then find an orthogonal vector to the two known directions, $[111]$ and $[\bar{1}11]$, which is $[0\bar{1}1]$ or $\hat{\mathbf{B}}$ in sample coordinates. Finally, we can find an orthogonal vector to $[0\bar{1}1]$ and $[111]$ again with the cross product, which is $[\bar{2}11]$ or $\hat{\mathbf{C}}$ in sample coordinates. The **UB** matrix is then determined by,

$$\mathbf{UB} = \begin{bmatrix} 1 & 0 & -2 \\ 1 & -1 & 1 \\ 1 & 1 & 1 \end{bmatrix}^{-1} \begin{bmatrix} \hat{\mathbf{A}} & \hat{\mathbf{B}} & \hat{\mathbf{C}} \end{bmatrix} \mathbf{B} \tag{S2}$$

Note that $\hat{\mathbf{A}}$, $\hat{\mathbf{B}}$, and $\hat{\mathbf{C}}$ are column vectors. Since the sample in this study has a FCC structure, **B** is the 3×3 identity matrix multiplied by the lattice constant at a specific time.

Burgers vector determination

Ordinarily, one cannot determine the Burgers vector with a single BCDI measurement. Often, multiple Bragg peaks are needed to test the invisibility criterion [2], meaning that dislocations are only visible when $\mathbf{Q}_{hkl}{\cdot}\mathbf{b} \neq 0$ [3]. As described in the main text, the possible Burgers vectors are $\mathbf{b} = \frac{a_0}{2}[110]$ or $\mathbf{b} = \frac{a_0}{2}[101]$. To select the Burgers vector, we compared ε_{111} to the theoretical strain field model, $\varepsilon_{111,\,\text{model}}$, calculated using each possible Burgers vector. See Experimental Section for the generation of the models. Here we note that austenitic SS is elastically anisotropic, but we anticipate that an isotropic model is sufficiently accurate to discern the correct Burgers vector. The 3D volume, $V_{\text{dislo.}}$, used for comparison is a pipe with a

30 nm radius surrounding the large dislocation, truncated by 10 dislocation nodes ($\approx$ 100 nm) at each end to prevent any grain boundary contributions. We masked ε_{111} values that exceeded the minimum 99.999th percentile of $|\varepsilon_{111}|$ ($\approx 6\times10^{-3}$) found in the measured and theoretical models. Figure S7 qualitatively compares the measured ε_{111} to each theoretical model using various slices through the large dislocation. It is difficult to determine which Burgers vector-generated $\varepsilon_{111,\text{ model}}$ matches better based on qualitative image comparisons.

Thus, a quantitative approach is to compute the χ^2 error and the Pearson correlation between each model and the measurement. The χ^2 error is defined as,

$$\chi^2 = \frac{\sum^{V_{\text{dislo.}}} (\varepsilon_{111} - \varepsilon_{111,\text{ model}})^2}{\sum^{V_{\text{dislo.}}} |\varepsilon_{111,\text{ model}}|} \tag{S3}$$

and the Pearson correlation is shown in Equation (8). Table S2 compares these error metrics for all the reconstructions before charging, which shows that all the reconstructions have a ε_{111} that matches closer to the $\varepsilon_{111,\text{ model}}$ for $\mathbf{b} = \frac{a_0}{2}[110]$ than for $\mathbf{b} = \frac{a_0}{2}[101]$. This is determined by the model that has a lower χ^2 and a higher r when computed against ε_{111}, giving confidence to $\mathbf{b} = \frac{a_0}{2}[110]$ as the correct Burgers vector for the large dislocation.

Dislocation elastic energy

The elastic energy of a mixed dislocation, E_{el} is given by [4],

$$E_{\text{el}} = \sum_{\xi} \frac{Gb^2(1 - \nu\cos^2\theta)}{4\pi(1-\nu)} \ln\left(\frac{R}{r_0}\right)(|\xi|) \tag{S4}$$

where $G = 77$ GPa, θ is the angle between $\mathbf{b}$ and dislocation segment ξ, R is the outside of the dislocation (here set to 30 nm), and r_0 is the inner cut-off radius ($|\mathbf{b}|$). The evolution of E_{el} and the dislocation line length is shown in Figure S9.

Dislocation simulation models

The determination of the correct Burgers vector was not trivial and certainly not conspicuous. With both $\frac{a_0}{2}[101]$ and $\frac{a_0}{2}[110]$ as candidates, simulations incorporating each Burgers vector were conducted. The results of two simulations are shown below in Figure S8: $\mathbf{b} = \frac{a_0}{2}[101]$/fixed surface nodes and $\mathbf{b} = \frac{a_0}{2}[110]$/fixed surface nodes. The descriptor "fixed surface nodes" refers to a simulation where the two end points of the large dislocation are stationary, which we also experimentally observed. In each simulation, the large dislocation moves to collapse into a

straight line. Given the absence of an external load, minimizing line length is expected, shown in Figure S9. Both simulations agree well with the experimental results, which suggests that this method alone cannot be used to identify the Burgers vector.

Estimate of beam-induced lattice strain

In addition to the experimental evidence that the X-ray beam does not influence our sample presented in the main text, here we provide a conservative, theoretical estimate that any beam-induced lattice strain on our measured grain is negligible. We used a 10 keV X-ray ($\approx 1.602 \times 10^{-15}$ J photon s^{-1}) with a flux of 5×10^9 photons s^{-1}. First we calculate the incident power of the X-ray, P,

$$P = \left(5 \times 10^9 \text{ s}^{-1}\right)\left(1.602 \times 10^{-15} \text{ J}\right) = 8 \times 10^{-6} \text{ J s}^{-1} = 8\ \mu\text{W}$$

During the experiment, there is a thin layer of ($\approx$ 0.1 mm) solution covering the HPT disk containing the grain, thus attenuating the X-rays. The fraction of the X-rays transmitted through the layer can be calculated using the Beer-Lambert law, and a linear attenuation coefficient, $\mu_l = 5.33$ cm^{-1} [5],

$$\text{Transmission} = e^{\mu_l x} = e^{\left(5.33 \text{ cm}^{-1}\right)(0.01 \text{ cm})} = 0.95$$

Therefore P_{incident} is,

$$P_{\text{incident}} = (8\ \mu\text{W})(0.95) = 7.6\ \mu\text{W}$$

SS has high thermal conductivity, $k_{\text{SS}} = 13.4$ W m^{-1} K^{-1}, and thermal diffusivity, $\alpha_{\text{SS}} = 3.48 \times 10^{-6}$ m^2 s^{-1}[6]. Although our beam is focused to a spot of size 810 nm × 860 nm ($\approx 7 \times 10^{-13}$ m^2), it is very small relative to the entire disk. Assuming the focused beam is a point source, the time for the energy to diffuse throughout the entire disk with a radius, r, of 2.5 mm is,

$$t \approx \frac{r^2}{\alpha_{\text{SS}}} = \frac{\left(2.5 \times 10^{-3} \text{ m}\right)^2}{3.48 \times 10^{-6} \text{ m}^2 \text{ s}^{-1}} = 1.8 \text{ s}$$

Thus, the X-ray energy is rapidly dispersed throughout the entire sample. Subsequently, the energy from the X-rays is dissipated by the surrounding solution. The solution is at 298 K and flows at 5 mL min^{-1} or $F = 8.33 \times 10^{-8}$ m^3 s^{-1} throughout the entire experiment, thus acting as

a heat sink. To determine the temperature rise of the disk, we need to estimate the heat transfer coefficient, h, between the SS disk and the water. To obtain a reasonable estimate requires multiple steps. First, we know that the solution flows over the area covered by the Kapton film (Figure S1), with a surface area, $SA = \pi\left(10^{-2}\text{ m}\right)^2 = 3.14 \times 10^{-4}\text{ m}^2$. The linear velocity is therefore,

$$v = \frac{F}{SA} = \frac{8.33 \times 10^{-8}\text{ m}^3\text{ s}^{-1}}{3.14 \times 10^{-4}\text{ m}^2} = 2.65 \times 10^{-4}\text{ m s}^{-1}$$

Next, we calculate the Reynold's number (Re) [6] to determine whether it's laminar or turbulent flow, where $\rho = 1000\text{ kg m}^{-3}$ is the density of water and $\mu = 8.9 \times 10^{-4}\text{ kg s}^{-1}\text{ m}^{-1}$ is the dynamic viscosity of water at 298 K [6],

$$Re = \frac{\rho v d}{\mu} = \frac{\left(1000\text{ kg m}^{-3}\right)\left(2.65 \times 10^{-4}\text{ m s}^{-1}\right)\left(5 \times 10^{-3}\text{ m}\right)}{8.9 \times 10^{-4}\text{ kg s}^{-1}\text{ m}^{-1}} = 1.5$$

The flow is laminar since $Re < 2300$ [6]. For laminar flow over a flat plate or disk, the Nusselt number (Nu) can be approximated using empirical correlations. For laminar flow, a common correlation is [6],

$$Nu \approx 0.664\left(Re^{1/2}\right)\left(Pr^{1/3}\right) = 0.664(1.5)^{1/2}(6)^{1/3} = 1.48$$

where $Pr \approx 6$ is the Prandtl number for water [6]. Using the Nusselt number, Nu, and $k_{\text{water}} = 0.61\text{ W m}^{-1}\text{ K}^{-1}$ we can determine the heat transfer coefficient for this system [6],

$$h = \frac{Nu k_{\text{water}}}{2r} = \frac{1.48 \times 0.61\text{ W m}^{-1}\text{ K}^{-1}}{2\left(2.5 \times 10^{-3}\text{ m}\right)} = 181\text{ W m}^{-2}\text{ K}^{-1}$$

The disk has a height, h, of 0.3 mm. The surface area, A, that makes contact with the solution is the top and wall of the disk,

$$A = \pi r^2 + 2\pi r h = \pi\left(2.5 \times 10^{-3}\text{ m}\right)^2 + 2\pi\left(2.5 \times 10^{-3}\text{ m}\right)\left(3 \times 10^{-4}\text{ m}\right) = 2.44 \times 10^{-5}\text{ m}^2$$

The mass, m, of the sample assuming a density, $\rho = 8238\text{ kg m}^{-3}$ [6], is,

$$m = \rho(\pi r^2 h) = \left(8238\text{ kg m}^{-3}\right)\left[\pi\left(2.5 \times 10^{-3}\text{ m}\right)^2\left(3 \times 10^{-4}\text{ m}\right)\right] = 4.85 \times 10^{-5}\text{ kg}$$

We can now calculate the thermal time constant, τ, assuming a heat capacity, $c_p = 468\text{ J kg}^{-1}\text{ K}^{-1}$ [6], is,

$$\tau = \frac{mc_p}{hA} = \frac{(4.85 \times 10^{-5}\ \text{kg})\ (468\ \text{J kg}^{-1}\ \text{K}^{-1})}{(181\ \text{W m}^{-2}\ \text{K}^{-1})\ (2.44 \times 10^{-5}\ \text{m}^2)} = 5.14\ \text{s}$$

This means the system will reach steady state at about $5\tau = 26$ s, which means that over the entire experiment, it is safe to assume a steady state heat transfer from the disk to the solution. We can now estimate the change in temperature, ΔT,

$$\Delta T = \frac{P_{\text{incident}}}{hA} = \frac{7.6 \times 10^{-6}\ \text{W}}{(181\ \text{W m}^{-2}\ \text{K}^{-1})\ (2.44 \times 10^{-5}\ \text{m}^2)} = 1.72 \times 10^{-3}\ \text{K}$$

Hence, the thermal strain, $\varepsilon_{\text{thermal}}$, calculated using the linear thermal expansion coefficient of 316 SS, $\alpha \approx 16 \times 10^{-6}\ \text{K}^{-1}$, is

$$\varepsilon_{\text{thermal}} = \alpha \Delta T = \left(16 \times 10^{-6}\ \text{K}^{-1}\right) \left(1.72 \times 10^{-3}\ \text{K}\right) = 2.75 \times 10^{-8}$$

Thus, a beam-induced strain on the order of $\approx 10^{-8}$ is negligible in the context of typical BCDI experiments, which has a strain sensitivity of $\approx 10^{-4}$. Heating would be nearly homogeneous throughout the volume of the grain, since it is overfilled in the X-ray spot and absorption is negligible across the depth. As an upper limit, let's assume that no conduction occurs between the sample and the surrounding solution. Now, we compute the temperature change caused by the heat accumulated in the sample over the entire experiment, which is about 15 h. The total energy input, Q, over $T = 15$ h, is,

$$Q = P_{\text{incident}} T = \left(7.6 \times 10^{-6}\ \text{W}\right) \left(5.4 \times 10^{4}\ \text{s}\right) = 0.410\ \text{J}$$

The temperature rise over the entire duration of the experiment is therefore given by,

$$\Delta T = \frac{Q}{mc_p} = \frac{0.410\ \text{J}}{(4.85 \times 10^{-5}\ \text{kg})\ (468\ \text{J kg}^{-1}\ \text{K}^{-1})} = 18.0\ \text{K}$$

We then compute the thermal strain assuming no conduction with the environment,

$$\varepsilon_{\text{thermal}} = \alpha \Delta T = \left(16 \times 10^{-6}\ \text{K}^{-1}\right) (18.0\ \text{K}) = 2.9 \times 10^{-4}$$

Thus, if our sample is instead isolated in a vacuum, a beam-induced thermal strain of $\approx 2.9 \times 10^{-4}$ can be resolved by the end of the experiment. Obviously, this is not the case here, and we expect a beam-induced thermal strain of $\approx 10^{-8}$ as outlined above, which has negligible impact on our sample and BCDI results.

Hydrogen diffusion into the grain

To investigate the diffusion of hydrogen into the austenitic 316 stainless steel grain, a one-dimensional diffusion model was developed using Fick's second law:

$$\frac{\partial C(y,t)}{\partial t} = D\frac{\partial^2 C(y,t)}{\partial y^2} \tag{S5}$$

where $C(y,t)$ is the hydrogen concentration, D is the diffusion coefficient, y is the position beneath the hydrogen-exposed surface, and t is time. The analytical solution to the diffusion equation, obtained via separation of variables, is:

$$C(y,t) = C_s - \frac{4C_s}{\pi}\sum_{n=0}^{N-1}\frac{1}{2n+1}\sin\left(\frac{(2n+1)\pi y}{2L}\right)\exp\left(-\frac{(2n+1)^2\pi^2 Dt}{4L^2}\right) \tag{S6}$$

where C_s is the constant surface concentration in mol L^{-1} during charging and $2L = 300$ μm is the thickness of the SS disk. We assume $D = 1.7 \times 10^{-16}$ m^2 s^{-1} for austenite [7]. The calculation was performed up to $N = 500$.

The model assumes hydrogen is introduced from the top and bottom surfaces of the disk, approximated as an infinite, homogeneous slab, under room temperature and atmospheric pressure. Thus, the boundary and initial constraints are:

- At $y = 0$, $C(0,t) = C_s$, where C_s is the constant surface concentration of hydrogen.
- $C(y,0) = 0$ for $0 < y < 2L$, assuming no hydrogen is initially present within the disk.

From Fig. 1b, the hydrogen concentration within the grain in appm, C_{appm}, is known as a function of time. To convert this to mol L^{-1}, we use the following equation,

$$C(y,t) = \frac{4C_{\text{appm}}(y,t)}{a_0^3 N_A} \times 10^{-9} \tag{S7}$$

where $N_A = 6.022 \times 10^{23}$ atoms mol^{-1} is Avogadro's number. From Eq. S6, and the hydrogen concentration data presented in Fig. 1b, we can estimate C_s and depth of the grain beneath the surface of the sample. By minimizing the squared error between the time evolution of hydrogen measured in the grain (Fig. 1b) and Eq. S7 using a Nelder-Mead simplex method [8], we obtain $C_s = 5.64 \pm 0.43$ mol L^{-1} or $3.96 \pm 0.30 \times 10^4$ appm and determine that the grain is 1.9 ± 0.2 μm beneath the surface (Fig. S3). The resulting hydrogen concentration profile as a function of time is shown in Fig. S10a.

The hydrogen concentration profile will create a corresponding volumetric strain profile, $\varepsilon_{\mathrm{vol}}(y,t)$, arising from the small lattice expansion induced by interstitial hydrogen,

$$\varepsilon_{\mathrm{vol}}(y,t) = \Omega_R C_{\mathrm{appm}}(y,t) \times 10^{-6} = \frac{\Omega_R a_0^3 N_A C(y,t)}{4} \times 10^3 \tag{S8}$$

where $\Omega_R = 0.2$ is the relaxation volume of hydrogen in FCC iron [9]. The resulting volumetric strain profile is shown in Fig. S10b.

Estimate of hydrogen-induced stresses and forces

The volumetric strain will induce internal stresses with equal magnitude in-plane and out-of-plane. The stress tensor, $\sigma_{\mathrm{internal}}$, is computed using the bulk modulus, K, defined as,

$$K = \frac{2G(1+\nu)}{3(1-2\nu)} \tag{S9}$$

where $G = 77$ GPa is the shear modulus and $\nu = 0.28$ is the Poisson's ratio. The resulting $\sigma_{\mathrm{internal}}$ is,

$$\sigma_{\mathrm{internal}}(y,t) = \begin{bmatrix} \frac{K}{3}\varepsilon_{\mathrm{vol}}(y,t) & 0 & 0 \\ 0 & \frac{K}{3}\varepsilon_{\mathrm{vol}}(y,t) & 0 \\ 0 & 0 & \frac{K}{3}\varepsilon_{\mathrm{vol}}(y,t) \end{bmatrix} \tag{S10}$$

The internal stress state is hydrostatic, indicating that there is no shear stress caused by lattice swelling alone.

In our sample, geometry-induced shear stresses may arise from the boundary constraint (b.c.) imposed by the as-yet largely hydrogen-free bulk of the sample. This as-yet uncharged bulk imposes that the in-plane strains in the hydrogen-charged layer must be zero. Thus hydrogen diffusion from the top surface of the disk will lead to an out-of-plane strain, $\varepsilon_{yy}(y,t)$. It is calculated as [10],

$$\varepsilon_{yy}(y,t) = \frac{\varepsilon_{\mathrm{vol}}(y,t)(1+\nu)}{3(1-\nu)} \tag{S11}$$

$\varepsilon_{yy}(y,t)$ can be projected onto the $[111]$ direction to obtain $\varepsilon_{111,\ \mathrm{b.c.}}(y,t)$,

$$\varepsilon_{111,\ \mathrm{b.c.}}(y,t) = \hat{\boldsymbol{n}}_{111}^{\mathsf{T}} \begin{bmatrix} 0 & 0 & 0 \\ 0 & \varepsilon_{yy}(y,t) & 0 \\ 0 & 0 & 0 \end{bmatrix} \hat{\boldsymbol{n}}_{111} \tag{S12}$$

where $\hat{\boldsymbol{n}}_{111}$ is the unit column vector for the $[111]$ direction. The $\varepsilon_{111,\,\text{b.c.}}(y,t)$ profiles are shown in Fig. S10c.

A comparison between $\varepsilon_{111,\,\text{b.c.}}$, the heterogeneous strain (ε_{111}), the heterogeneous strain generated by the dislocation model ($\varepsilon_{111,\,\text{model}}$), and the homogeneous strain (e_{111}) is shown in Fig. S11.

A zero in-plane strain boundary condition will generate an in-plane elastic stress, $\sigma_{\text{b.c.}}(y,t)$,

$$\sigma_{\text{b.c.}}(y,t) = \begin{bmatrix} \frac{-2G(1+\nu)}{3(1-\nu)}\varepsilon_{\text{vol}}(y,t) & 0 & 0 \\ 0 & 0 & 0 \\ 0 & 0 & \frac{-2G(1+\nu)}{3(1-\nu)}\varepsilon_{\text{vol}}(y,t) \end{bmatrix} \tag{S13}$$

This in-plane elastic stress can be applied to the dislocation dynamics model for comparison to the experimental data. The results are shown in Fig. S12.

The in-plane elastic stress also generates a resolved shear stress $\tau_{\text{b.c.}}$, on the $(\bar{1}11)$ glide plane in the $[110]$ Burgers vector direction, which can be computed by,

$$\tau_{\text{b.c.}}(y,t) = \hat{\boldsymbol{b}}_{110} \cdot \sigma_{\text{b.c.}}(y,t)\hat{n}_{\bar{1}11} \tag{S14}$$

The resulting resolved shear stress profile is shown in Fig. S10d.

The $\tau_{\text{b.c.}}$ can be used to calculate the Peach–Koehler force on the large dislocation,

$$\boldsymbol{F}_{\text{b.c.}}(y,t) = (\sigma_{\text{b.c.}}(y,t) \cdot \boldsymbol{b}_{110}) \times \hat{\xi} \tag{S15}$$

where $\hat{\xi}$ is the dislocation line direction. We can compute the local $\boldsymbol{F}_{\text{b.c.}}(y,t)$ for each dislocation segment in the large dislocation at different times. The resulting map is shown in Fig. S13.

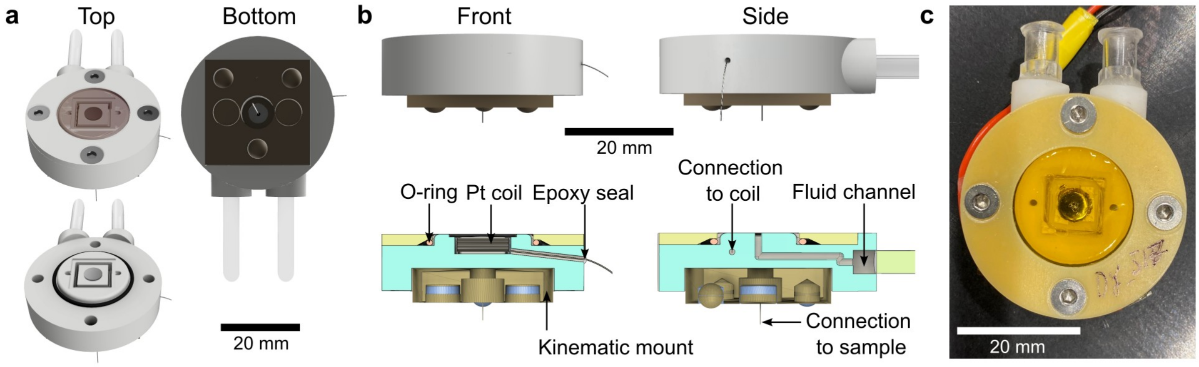

Figure S1: Details of the bespoke electrochemical flow cell. a) Top and bottom views of the flow cell. The lid and the brown Kapton film are removed in the bottom left inset, exposing the HPT disk and O-ring. b) Front and side outer views of the flow cell in the top row, with cross-sections in the bottom row. c) Photograph of the flow cell with the sample loaded.

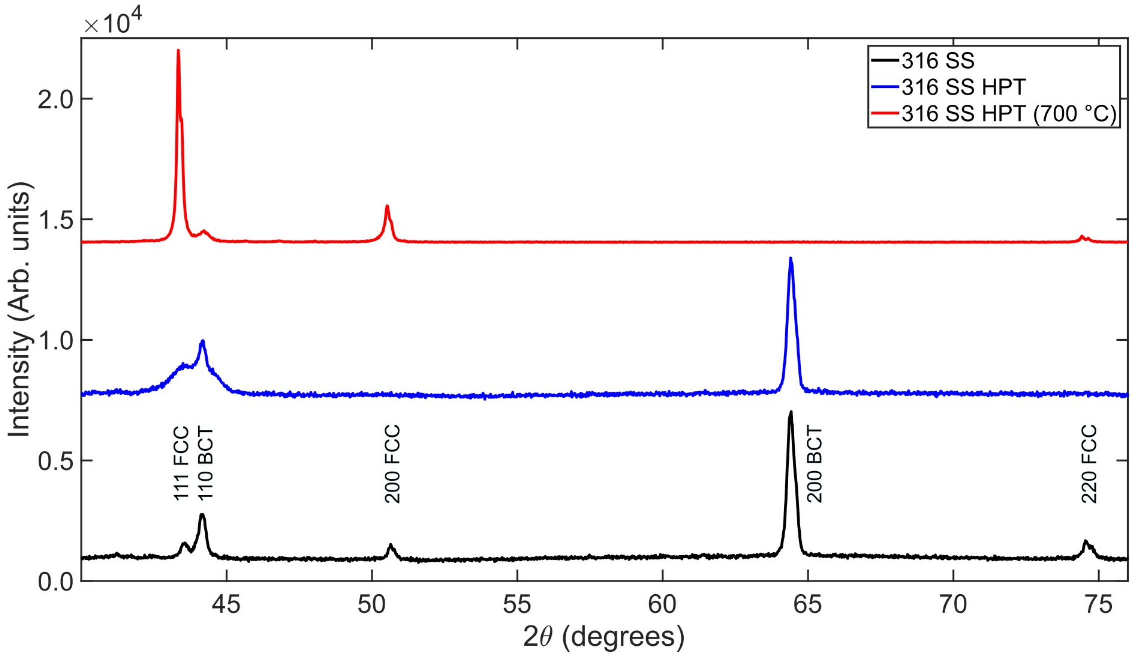

Figure S2: Powder X-ray diffraction of a 316 SS sample through different processing stages using a Cu Kα source. The as-received sample (black), contains both FCC and body-centered tetragonal (BCT) phases. After HPT (blue), the sample is predominantly BCT, which then transforms into predominantly FCC after annealing (red).

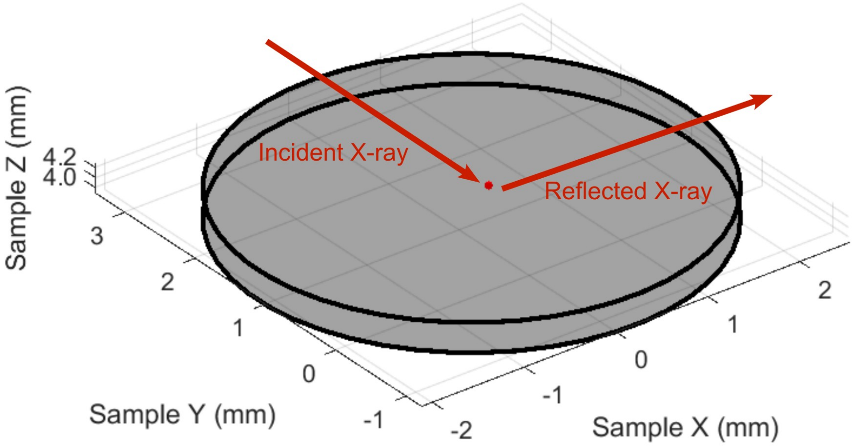

Figure S3: The grain position (red dot) relative to the SS disk. The center of the grain is located 1.9 ± 0.2 μm beneath the surface based on our hydrogen diffusion model. Note the position of the disk is defined by the sample translation stage motors in the laboratory coordinate frame at reference sample rotation angles, which is different to the hydrogen diffusion model.

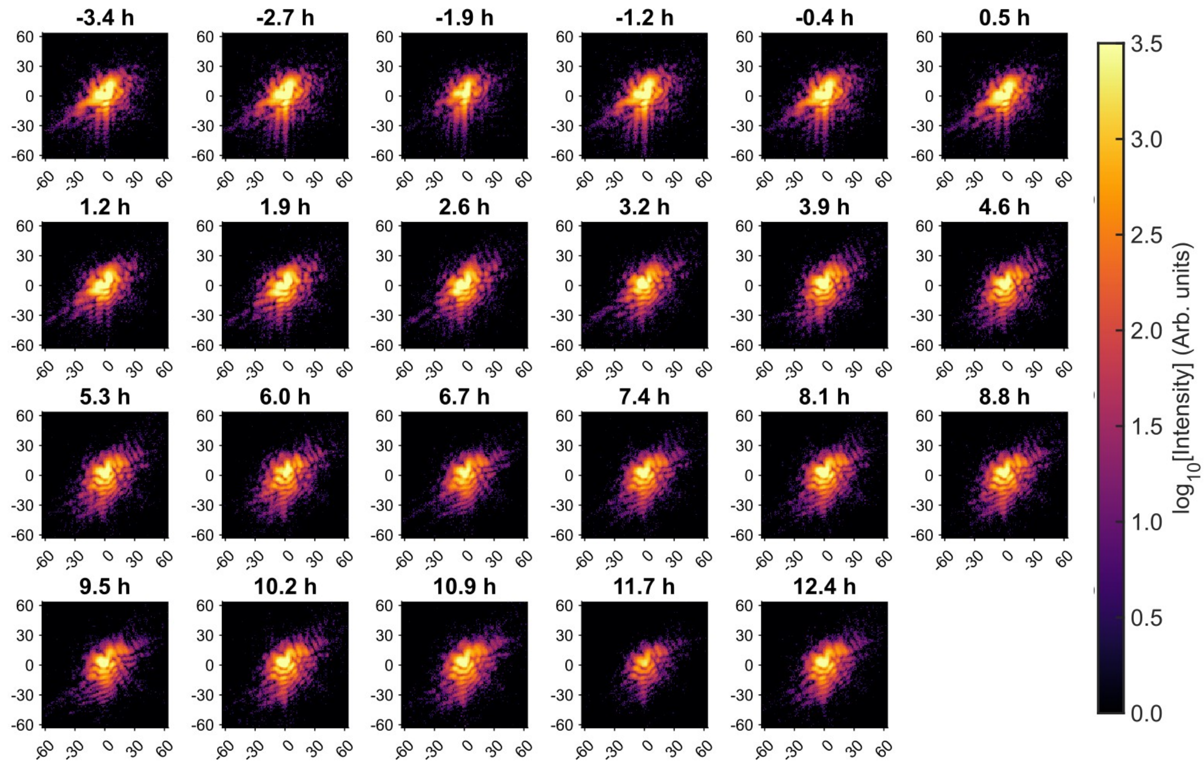

Figure S4: Slices through the center of mass of the 111 Bragg peaks. Images are shown in detector pixel coordinates (unbinned) relative to the center of the peak. The array is cropped to a size of 128 × 128 pixels.

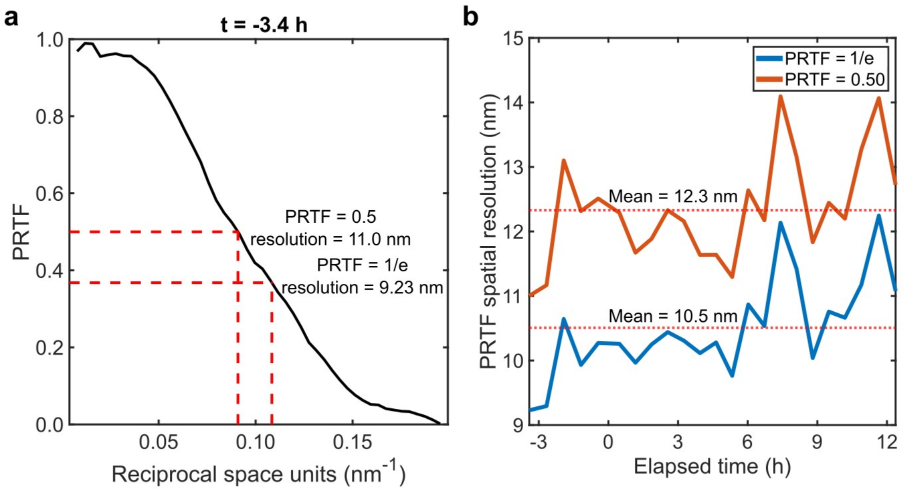

Figure S5: Spatial resolution determined using the PRTF. a) An example of determining the spatial resolution at –3.4 h using the PRTF for a cutoff of 0.50 and $1/e$. b) The spatial resolutions for each reconstruction. The dotted lines show the mean for each PRTF cutoff.

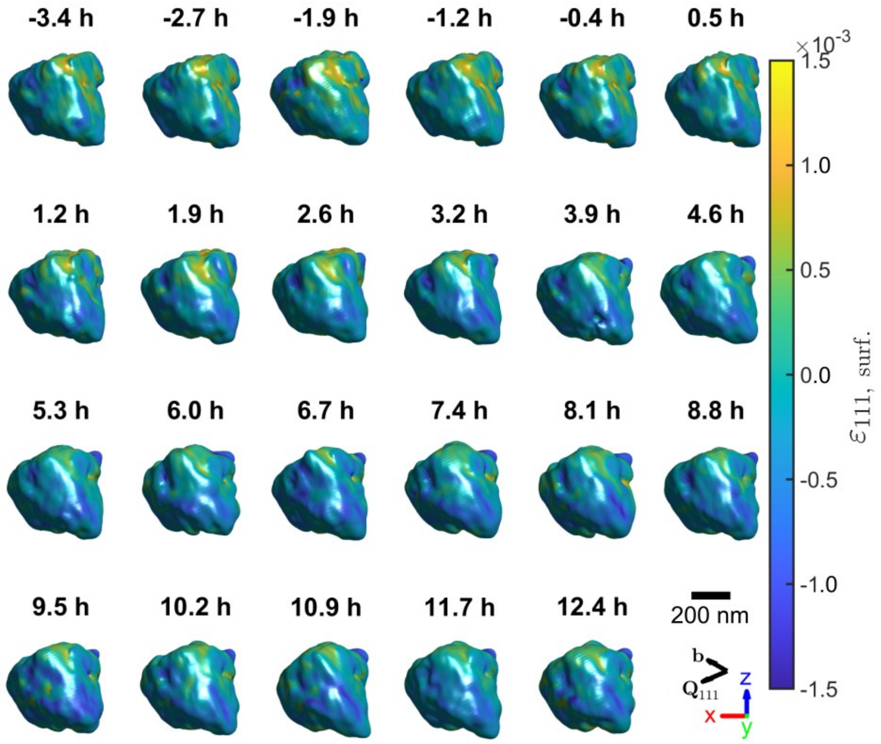

Figure S6: Evolution of $\varepsilon_{111,\text{ surf.}}$ before and during hydrogen charging for all reconstructions. The amplitude threshold was set for each reconstruction according to the Supporting Information. See Video S1 for additional views.

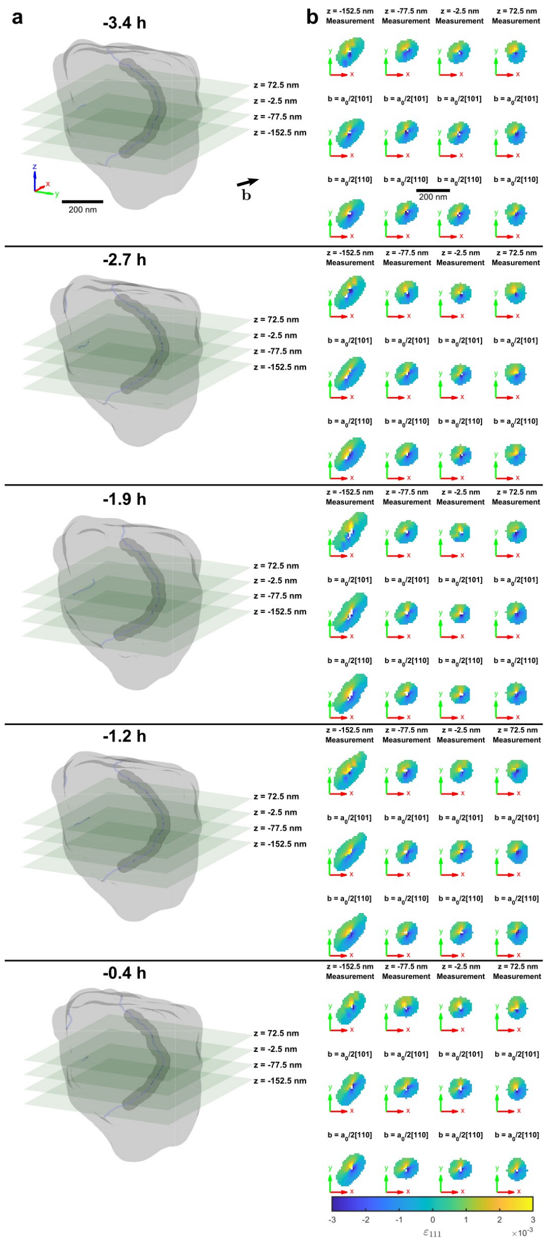

Figure S7: Comparison of slices through ε_{111} and $\varepsilon_{111,\ \text{model}}$ surrounding the dislocation before hydrogen charging. a) A translucent rendering of the grain morphology with the dislocations at different times. A mask surrounding the central portion of the dislocation is shown as a dark region. Slices at various points along the z-axis are shown as green planes. b) ε_{111} values within the mask for each reconstruction, at the positions indicated by the green planes in a). Voxels at the dislocation core exceeding the threshold are masked. The measurement is compared to the theoretical models computed using each possible Burgers vector.

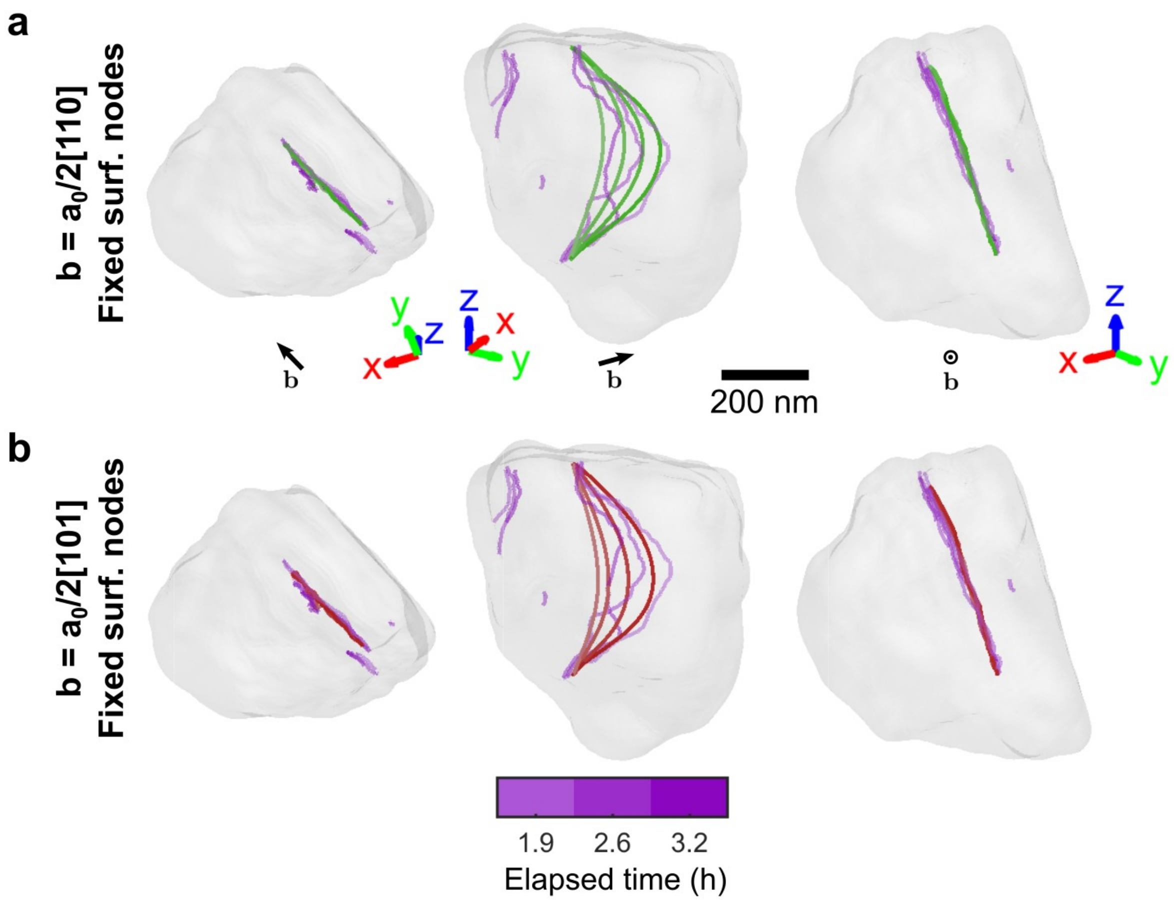

Figure S8: Comparison of dislocation dynamics simulation models for the large dislocation. a) The simulation using $\mathbf{b} = \frac{a_0}{2}[110]$ (green). b) The simulation using $\mathbf{b} = \frac{a_0}{2}[101]$ (red). Both rows show a translucent rendering of the grain morphology with the dislocations during glide (purple) between 1.9 h to 3.2 h, as presented in Figure 3. The large dislocation is initially bow-shaped and straightens out (becomes shorter) as time progresses. The first row is included in Fig 3.

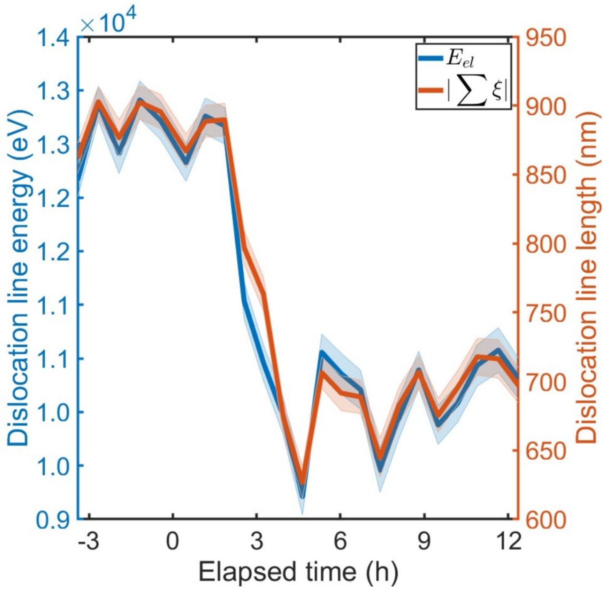

Figure S9: The evolution of the elastic energy and length of the large dislocation. The shaded areas represent the error dictated by the spatial resolution for each reconstruction.

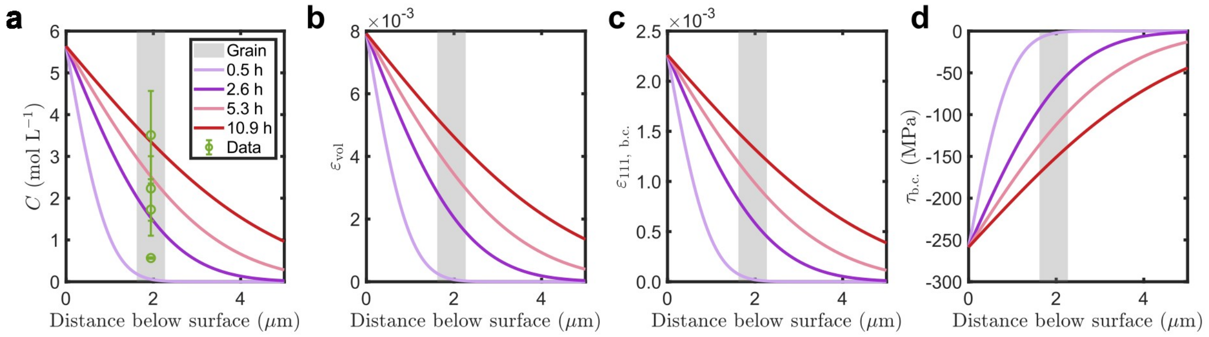

Figure S10: The evolution of hydrogen concentration and its associated strains and stresses based on a 1D Fick's second law of diffusion model through the SS disk. The following is shown as a function of depth for select times: a) hydrogen concentration (Eq. S6), b) volumetric strain (Eq. S8), c) out-of-plane strain generated by boundary constraints projected onto the [111] direction (Eq. S12), and d) resolved shear stress due to boundary constraints (Eq. S14). The stress is computed on the large dislocation with a $\frac{a_0}{2}[110]$ Burgers vector and sits on a $(\bar{1}11)$ glide plane. The shaded region is the position of the grain. The data in a) is obtained from Fig. 1b.

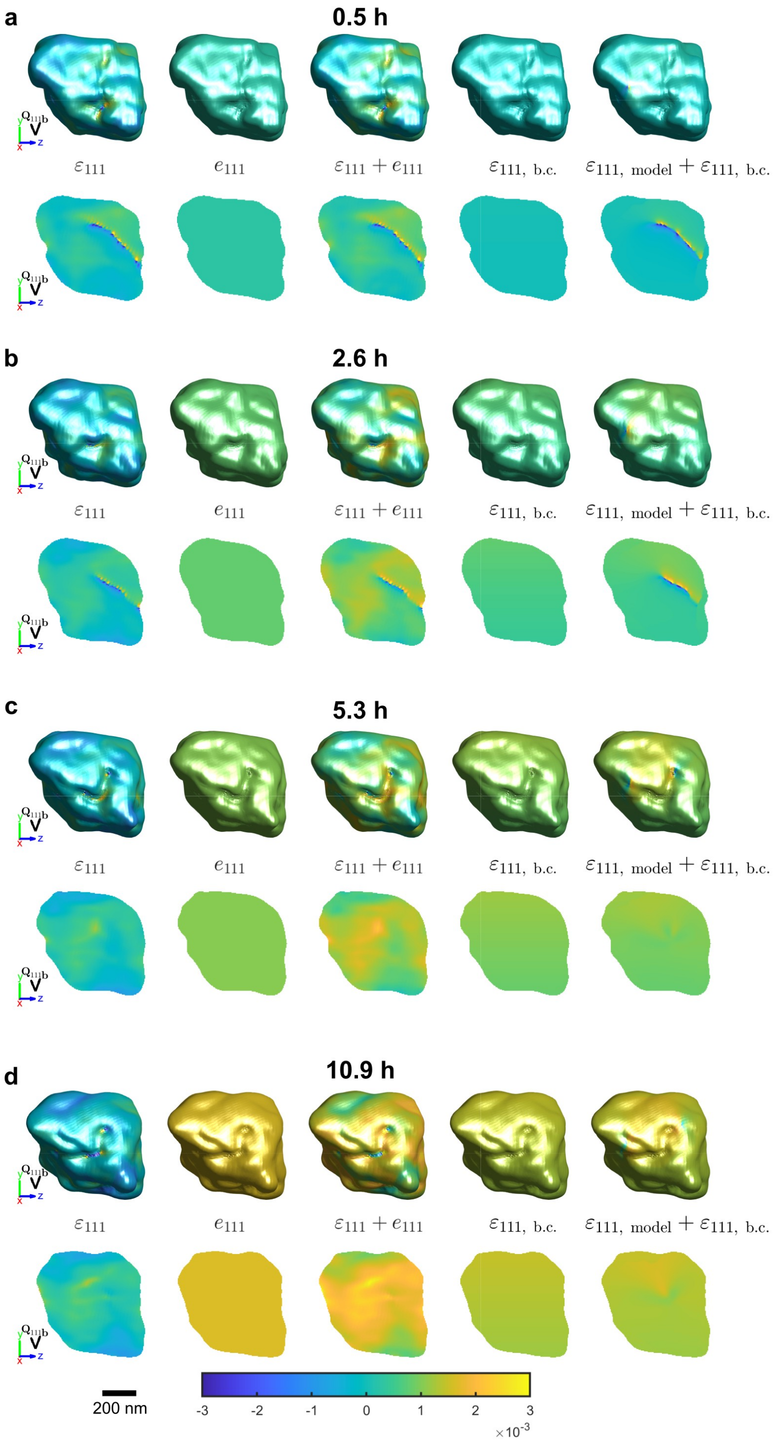

Figure S11: Comparison between the heterogeneous strain (ε_{111}), the homogeneous strain (e_{111}), the boundary condition-induced strain ($\varepsilon_{111,\ \mathrm{b.c.}}$), and the heterogeneous strain generated by the elastic dislocation model ($\varepsilon_{111,\ \mathrm{model}}$) during hydrogen charging. 3D images of the reconstruction and $y - z$ slices at the center of the grain are shown at select times: a) 0.5 h, b) 2.6 h, c) 5.3 h, and d) 10.9 h. The scale bar and color bar apply to the entire figure.

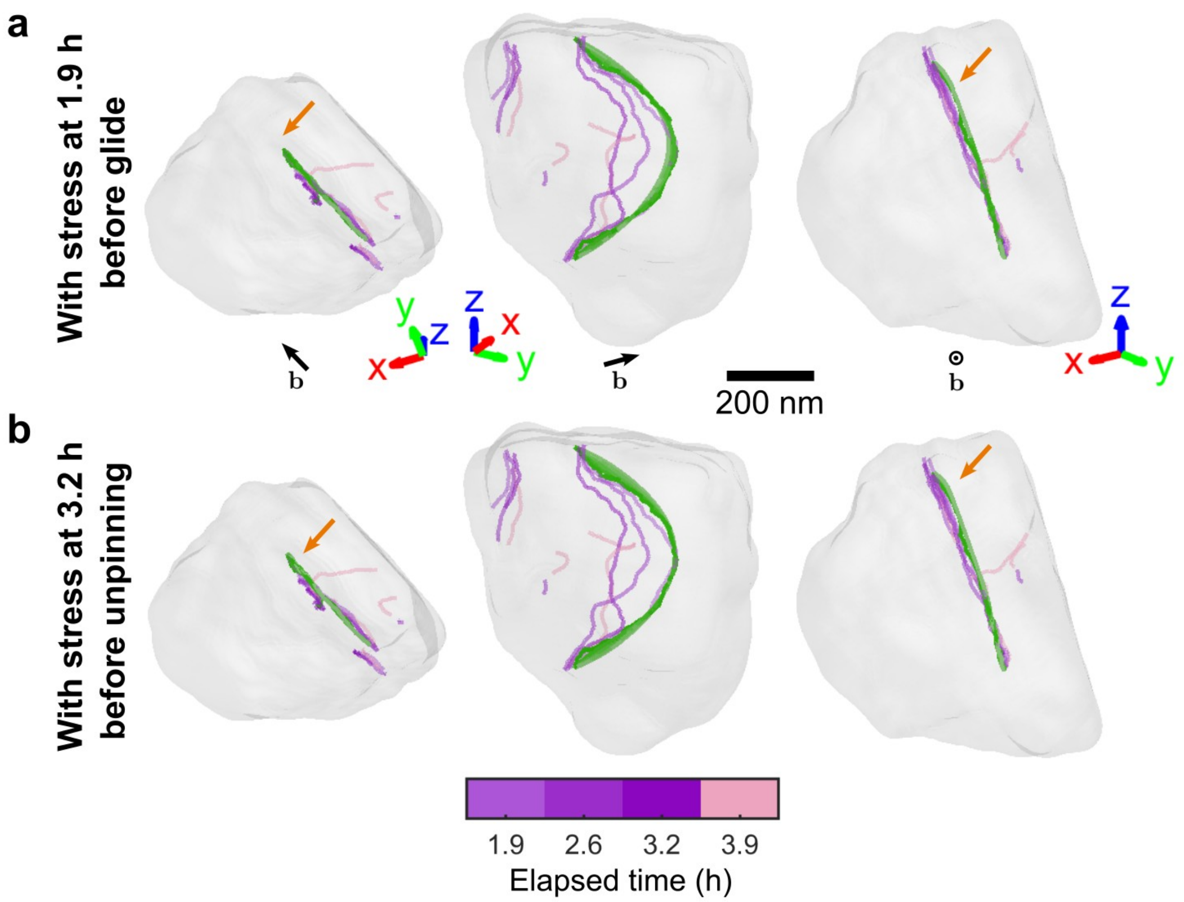

Figure S12: Comparison of experimental data to dislocation dynamics simulation models with stresses from hydrogen concentration gradients. The dislocation dynamics model with $\mathbf{b} = \frac{a_0}{2}[110]$ (green) follows the model presented in Figure 3, but with applied stresses at onset of dislocation movement: a) 1.9 h and b) 3.2 h. Both rows show a translucent rendering of the grain morphology, with the dislocation during glide (purple) from 1.9 h to 3.2 h, followed by unpinning and climb (pink) at 3.9 h. The orange arrow indicates where the dislocation dynamics model appears to be slightly lifted in the direction of unpinning.

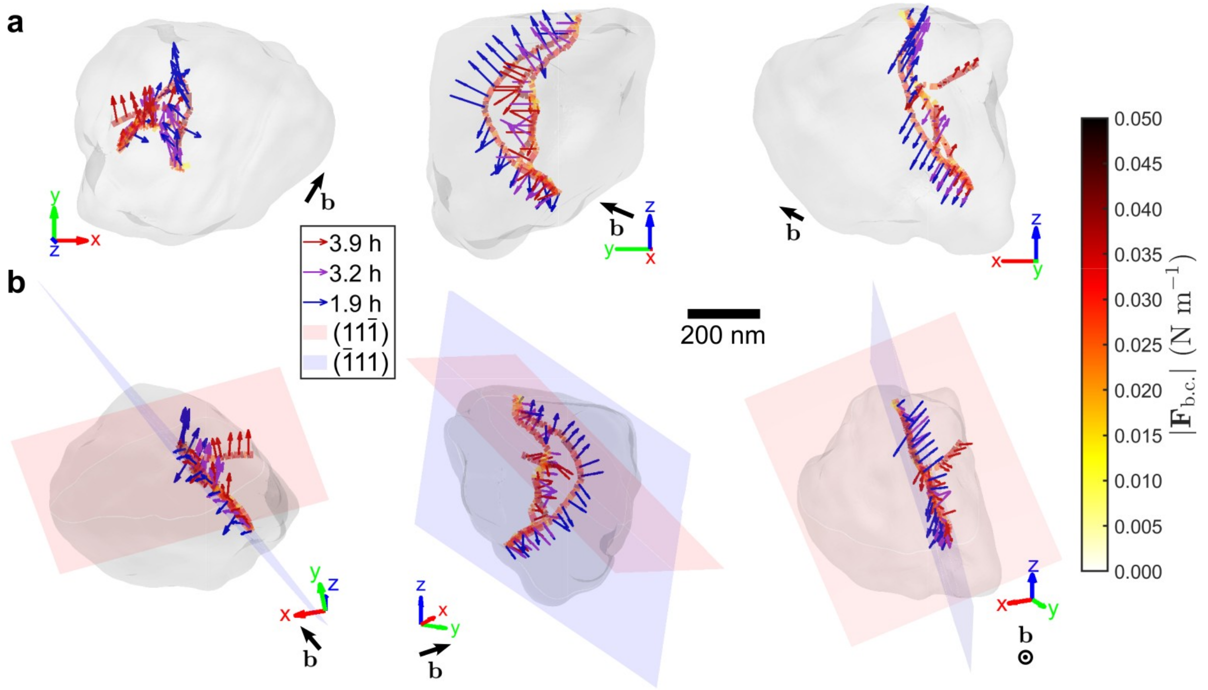

Figure S13: Dislocation glide, unpinning, and climb. A translucent isosurface of the grain showing only the large dislocation at three different times indicated by the Peach–Koehler force vectors (Eq. S15), which are shown for every third dislocation node for clarity. Each segment joining two dislocation nodes is colored by the Peach–Koehler force magnitude. The black arrow indicates the Burgers vector direction. a) Orthogonal views based on sample coordinates. b) Crystallographic views based on the dislocation loop plane, with the dislocation initially lying on the $(\bar{1}11)$ plane, and later one end unpins and climbs onto the $(11\bar{1})$ plane.

Table S1: Elemental composition of 316 SS (wt%). Data is from RS components technical datasheet (stock number 264-7241). Composition is related to the following specifications: AS2837-1986-316, AISI TYPE 316, and ASTM A276-316.

C	Ni	Cr	Mo
0.08	12.0	17	2.25

Table S2: Comparison of χ^2 error and r between ε_{111} and $\varepsilon_{111,\text{ model}}$ values before charging. The shaded cell indicates the expected Burgers vector, based on a lower χ^2 error and a greater r value.

Elapsed time (h)	**b** model	χ^2 error ($\times 10^{-4}$)	r
−3.4	$\frac{a_0}{2}[101]$	5.57	0.790
	$\frac{a_0}{2}[110]$	5.13	0.806
−2.7	$\frac{a_0}{2}[101]$	6.15	0.762
	$\frac{a_0}{2}[110]$	5.54	0.786
−1.9	$\frac{a_0}{2}[101]$	5.88	0.766
	$\frac{a_0}{2}[110]$	5.59	0.778
−1.2	$\frac{a_0}{2}[101]$	5.45	0.781
	$\frac{a_0}{2}[110]$	4.81	0.806
−0.4	$\frac{a_0}{2}[101]$	5.53	0.785
	$\frac{a_0}{2}[110]$	4.91	0.808

Caption for Video S1 Orbits around all the reconstruction isosurfaces, colored by $\varepsilon_{111,\ \mathrm{surf.}}$**.** The starting view is along $[1\bar{1}2]$, consistent with the other Videos. The directions of $\mathbf{Q}_{111}$ and $\mathbf{b} = \frac{a_0}{2}[110]$ are indicated using black arrows. The coordinate axis lengths have a magnitude of 200 nm.

Caption for Video S2 Orbits around the dislocations for all reconstructions. The starting view is along $[1\bar{1}2]$, consistent with the other Videos. The directions of $\mathbf{Q}_{111}$ and $\mathbf{b} = \frac{a_0}{2}[110]$ are indicated using black arrows. The coordinate axis lengths have a magnitude of 200 nm.

Caption for Video S3 Slices through the internal ε_{111} **for all reconstructions along** $[1\bar{1}2]$**, perpendicular to the Burgers vector.** The x, y and z coordinates in the video title correspond to a line drawn through the grain at the center when viewed along $[1\bar{1}2]$. The directions of $\mathbf{Q}_{111}$ and $\mathbf{b} = \frac{a_0}{2}[110]$ are indicated using black arrows. The coordinate axis lengths have a magnitude of 200 nm.

References

[1] H. N. Chapman, A. Barty, S. Marchesini, A. Noy, S. P. Hau-Riege, C. Cui, M. R. Howells, R. Rosen, H. He, J. C. H. Spence, U. Weierstall, T. Beetz, C. Jacobsen, D. Shapiro, *Journal of the Optical Society of America A* **2006**, *23* 1179.

[2] F. Hofmann, N. W. Phillips, S. Das, P. Karamched, G. M. Hughes, J. O. Douglas, W. Cha, W. Liu, *Physical Review Materials* **2020**, *4* 013801.

[3] D. B. Williams, B. C. Carter, *Transmission Electron Microscopy: A Textbook for Materials Science*, Springer, 2 edition, **2009**.

[4] P. M. Anderson, J. P. Hirth, J. Lothe, *Theory of Dislocations*, Cambridge University Press, 3 edition, **2017**.

[5] J. H. Hubbell, S. Seltzer, X-ray mass attenuation coefficients, **2004**.

[6] F. P. Incropera, D. Dewitt, T. L. Bergman, A. Lavine, *Fundamentals of Heat and Mass Transfer*, John Wiley, 6 edition, **2007**.

[7] I. M. Robertson, H. K. Birnbaum, P. Sofronis, *Chapter 91 Hydrogen Effects on Plasticity*, volume 15, 249–293, Elsevier, ISBN 1572-4859, **2009**.

[8] J. C. Lagarias, J. A. Reeds, M. H. Wright, P. E. Wright, S. J. Optim, *SIAM Journal of Optimization* **1998**, *9* 112.

[9] D. G. Ulmer, C. J. Altstetter, *Acta Metallurgica et Materialia* **1993**, *41* 2235.

[10] F. Hofmann, D. Nguyen-Manh, M. R. Gilbert, C. E. Beck, J. K. Eliason, A. A. Maznev, W. Liu, D. E. Armstrong, K. A. Nelson, S. L. Dudarev, *Acta Materialia* **2015**, *89* 352.
